# Relationships between Personality Traits, Medial Temporal Lobe Atrophy, and White Matter Lesion in Subjects Suffering from Mild Cognitive Impairment

**DOI:** 10.3389/fnagi.2014.00195

**Published:** 2014-07-29

**Authors:** Emmanuelle Duron, Jean-Sébastien Vidal, Samira Bounatiro, Sana Ben Ahmed, Marie-Laure Seux, Anne-Sophie Rigaud, Olivier Hanon, Cécile Viollet, Jacques Epelbaum, Guillaume Martel

**Affiliations:** ^1^Service de Gériatrie, Hôpital Broca, Assistance Publique – Hôpitaux de Paris, Paris, France; ^2^Université Paris Descartes, Sorbonne Paris Cité, Equipe d’Accueil 4468, Paris, France; ^3^UMRS 894 INSERM, Centre de Psychiatrie et Neurosciences, Université Paris Descartes, Sorbonne Paris Cité, Paris, France

**Keywords:** conscientiousness, neuroticism, Alzheimer, mild cognitive impairment, medial temporal lobe, WML, leukoaraiosis

## Abstract

Mild cognitive impairment (MCI) is a heterogeneous cognitive status that can be a prodromal stage of Alzheimer’s disease (AD). It is particularly relevant to focus on prodromal stages of AD such as MCI, because patho-physiological abnormalities of AD start years before the dementia stage. Medial temporal lobe (MTL) atrophy resulting from AD lesions and cerebrovascular lesions [i.e., white matter lesions (WML), lacunar strokes, and strokes] are often revealed concurrently on magnetic resonance imaging (MRI) in MCI subjects. Personality changes have been reported to be associated with MCI status and early AD. More specifically, an increase in neuroticism and a decrease in conscientiousness have been reported, suggesting that higher and lower scores, respectively, in neuroticism and conscientiousness are associated with an increased risk of developing the disease. However, personality changes have not been studied concomitantly with pathological structural brain alterations detected on MRI in patients suffering from MCI. Therefore, the objective of the present study was to assess the relationship between MTL atrophy, WML, lacunar strokes, and personality traits in such patients. The severity of WML was strongly associated with lower levels of conscientiousness and higher levels of neuroticism. Conversely, no association was detected between personality traits and the presence of lacunar strokes or MTL atrophy. Altogether, these results strongly suggest that personality changes occurring in a MCI population, at high risk of AD, are associated with WML, which can induce executive dysfunctions, rather than with MTL atrophy.

## Introduction

Mild cognitive impairment (MCI) is a heterogeneous cognitive status, which can be a prodromal stage of Alzheimer’s disease (AD) (Petersen et al., 2001). MCI status is characterized by cognitive deficit in single or multiple domains without any impairment of activities of daily living. The risk of progression to dementia is 10–15% per year (Gauthier et al., 2006) but amnestic MCI has higher risk to progress to AD (Petersen, 2004). AD is the most frequent neurodegenerative disease leading to a devastating loss of autonomy (Ballard et al., 2011). To date, no curative treatment is available and therefore considering possible prodromal stages of disease such as MCI status is particularly relevant. Main neuropathological hallmarks of AD include amyloid plaques and neurofibrillary tangles leading to neuronal loss reflected by medial temporal lobe (MTL) atrophy. The latter spans hippocampus and amygdala regions, and it starts years before the clinical stage of dementia including the MCI period (Burton et al., 2009; Aisen et al., 2011). In the elderly, AD lesions are usually mixed with cerebrovascular lesions [white matter lesions (WML), lacunar strokes and strokes] and form the mixed dementia (Pantoni, 2002; Zekry et al., 2002). Similarly, these types of lesions are often mixed in MCI, which progress to dementia (Moorhouse and Rockwood, 2008). Since cerebrovascular lesions have been consistently found associated with AD lesions, they are considered to be substantial contributors to the development of cognitive impairment and dementia by themselves or by their synergistic effects on the pathogenesis associated with AD (for review, Roh and Lee, 2014). In addition, many longitudinal studies have shown that vascular risk factors (hypertension, diabetes, high cholesterol level, smoking, and alcohol) are risk factors for both cognitive impairment and AD (for review, Duron and Hanon, 2008) and for cerebrovascular lesions (Launer, 2004). For each vascular risk factor, a proper patho-physiological explanation can rationalize the association with AD [e.g., hypertension is associated with hippocampal atrophy independently of cerebrovascular lesions (Korf et al., 2004)]. However, association between vascular risk factors and AD can also depend on the summation of AD and cerebrovascular lesions, which may contribute to the early expression of sub-clinical AD reaching earlier the threshold of dementia (Duron and Hanon, 2008; Godin et al., 2010).

Personality is composed of a set of emotional qualities that characterize and define each individual. Their biological bases are determined by genetics (McCrae et al., 2000) as well as environmental processes (Caspi and Bem, 1990). According to Cloninger’s theory, there are two sides in personality, temperament, and character. Temperament is defined by percept-based habits and skills, while character is defined by concept-based goals and values (Cloninger, 1994). Temperament and character interact to create the unique personality of an individual. Among the different models developed to describe personality, the big-five model (BFM; Goldberg, 1990) also called the five-factor model (Costa and McCrae, 1987) is the most used. The five factors defined by this model are openness to experience, conscientiousness, extraversion, agreeableness, and neuroticism. The personality of each individual is defined by a score in each of the five BFM dimensions. Studies using this model show that personality is stable through years (Costa and McCrae, 1993, 2006; Costa et al., 2000; Weiss et al., 2005). Personality traits can be altered by structural brain changes such as the one resulting from neurological diseases (Wang et al., 2009; Duberstein et al., 2011). Indeed, the score in some personality traits is associated with cognitive impairment and AD in transversal (Duchek et al., 2007) and prospective studies (Wang et al., 2009; Duberstein et al., 2011). More specifically, two personality traits seem particularly relevant for AD, neuroticism, and conscientiousness (Robins Wahlin and Byrne, 2011). They are consistently altered in AD (for review, see Robins Wahlin and Byrne, 2011) and scores in these two dimensions of personality could discriminate between healthy aging patients and early-stage of AD (Duchek et al., 2007). In addition, a recent study reports consistent personality modifications in MCI subjects compared to control subjects (Donati et al., 2013). However, the relationship between personality traits and structural brain changes, either resulting from cerebrovascular diseases or AD pathology, is not documented in patients with cognitive impairment. Therefore, the objective of the present study was to assess the relationship between personality traits and brain structural changes (i.e., MTL atrophy, WML, and lacunar stroke) in patients with MCI.

## Methods

### Sample

Ninety subjects were recruited at a geriatric memory center from April to July 2013. Inclusion criteria included age being above 65 years, French speaking, with a brain magnetic resonance imaging (MRI) performed within a year and diagnosed as MCI according to the European AD Consortium [Peterson’s criteria (Portet et al., 2006)]. The different MCI subtypes [i.e., amnestic versus non-amnestic and single versus multiple domain (Petersen, 2004)] were not studied separately.

Non-inclusion criteria were neurological diseases that could interfere with cognition (epilepsy and Parkinson’s disease), disability that could bias cognitive tests (e.g., severe visual impairment), and major depressive state [according to DSMIV-R criteria (Wilson and Skodol, 1994)].

All data were anonymized prior to the study. All participants gave their consent for being anonymously included in our clinical research database. Written informed consents were available, and no review from the local institutional review board was needed.

## Measures

### Variables

#### Personality traits

The Big-Five Inventory [BFI-fr (Plaisant, 2010)] is a 45-item-self-report questionnaire with 8 items measuring each of the 5 domains constituting the BFM: neuroticism (e.g., “I often feel inferior to others”), Extraversion (e.g., “I like to have a lot of people around me”), Openness to Experience (e.g., “I have a lot of intellectual curiosity”), Agreeableness (e.g., “I try to be courteous to everyone I meet”), and Conscientiousness (e.g., “I keep my belongings clean and neat”). Response options comprise a five-point Likert scale from Strongly Disagree to Strongly Agree. Internal consistency (Cronbach’s coefficient alpha) for the five scales ranged from 0.70 (conscientiousness) to 0.76 (openness) in the current study. The BFM has been extensively studied and has been previously used in gerontological research (for review, see Robins Wahlin and Byrne, 2011), supporting its reliability and applicability in elderly.

#### MRI measurements

Magnetic resonance imaging protocols included axial and coronal T1, axial and coronal FLAIR T2, axial EG T2, and proton density T2 weighted images in a 1.5 T MR unit. Atrophy of the right and left MTL was visually assessed by trained neuro-radiologists (on coronal slices of 1.5 mm with no interslice gap) according to Scheltens’ scale (0, no atrophy; 1, light; 2, mild; 3, moderate; and 4, severe) (Korf et al., 2004). MTL atrophy stage was defined as the worst atrophy score (right or left). Lacunar strokes were defined on MRI as cavities with a diameter of 3–15 mm with signal intensities similar to cerebrospinal fluid in Flair, T1, and T2 scan sequences. Periventricular WML and subcortical WML were evaluated according to Fazekas criteria (0–3 scale, with higher scores indicating more severe WML) (Wahlund et al., 2001). Because only 6 subjects (out of 90) did not display any periventricular WML (grad-0), they were pooled with participants presenting slight periventricular WML (grad-1). Similarly, because only 5 subjects (out of 90) displayed severe subcortical WML (grad 3), they were pooled with participants presenting moderate subcortical WML (grad-2).

### Covariates

All subjects underwent cognitive assessment using the Mini Mental State Examination [MMSE (maximum score 30 points)], a validated and reliable tool to assess global cognitive function (Folstein et al., 1975). The educational level was scored as elementary school, secondary school, and high-school diploma and above. Vascular risk factors (hypertension, diabetes, high cholesterol level, smoking, and alcohol) were evaluated since they are risk factors for both AD and cerebrovascular lesions. Diabetes was defined as a history of diabetes, use of glucose-modifying medication, or fasting blood glucose ≥7 mmol/L. Hypertension was defined as measured systolic blood pressure ≥140 mmHg or diastolic blood pressure ≥90 mmHg, or self-reported history of hypertension, or using antihypertensive medications. Hypercholesterolemia was defined as history of dyslipidemia or use of lipid-lowering medication. Considering tobacco consumption, participants were classified into two groups according to the question “have you ever smoke?”: never smoker or ever smoker (which include current and former smoker). For alcohol consumption, at risk consummation was considered (i.e., three glasses of wine per day for woman and four for man). As migraine is also reported to be a risk factor for cerebrovascular lesions (Hougaard et al., 2014), an evaluation of migraine episodes was also performed.

### Statistical analysis

Data were analyzed using JMP Pro (Version 10, SAS Institute) and R (R Foundation for Statistical Computing, Vienna, Austria). Significant associations or differences (*p* value below 0.05) were highlighted by the use of bold in tables.

First, general characteristics of the overall population were expressed as mean and standard deviation for continuous variables and as number of subjects and percentage for categorical variables.

Second, relationships between different BFI items and continuous general characteristics were analyzed with Kendall’s Tau-b correlation coefficient, and relationships between different BFI items and categorical variables were analyzed with means and standard deviations and compared with analysis of variance.

Third, in order to take into account the correlation between the different BFI items, we performed mixed models that included the different BFI items as dependent variables and the MTL atrophy, subcortical WML, periventricular WML, or lacunar stroke separately as independent variables with adjustment for age, sex, and education level. In this model, the different BFI scores were standardized.

Fourth, models were fully adjusted with age, sex, and education level and lacunar stroke, MTL atrophy, subcortical WML, and periventricular WML simultaneously.

Finally, as sensitivity analyses, we further adjusted the fully adjusted models for the cardiovascular risk factors univariately associated with personality traits. All the mixed models were also run with the different BFI scores standardized by gender in order to take into account the difference in mean BFI between men and women.

## Results

General characteristics of the 90 included participants are presented in Table 1. Mean age of the sample was 80.0 (6.1) years, with 62 (68.9%) women. The mean MMSE score was 27.0 (2.2). Fifty-six participants (62.2%) graduated from high school, 24 participants (26.8%) reached secondary school, and 10 participants (11.0%) stopped after elementary school. In 15 participants (16.7%), the presence of at least one lacunar stroke was detected. For MTL atrophy, 19 participants (21.1%) did not show any atrophy or only light atrophy, 29 participants (32.2%) displayed mild atrophy, 33 participants (36.7%) displayed moderate atrophy, and 9 participants (10.0%) showed severe atrophy. Twenty-two participants (24.4%) had no subcortical WML, 43 participants (47.8%) had mild, and 25 participants (27.8%) moderate to severe subcortical WML. Finally, for periventricular WML, 44 participants (48.9%) had no lesion or mild lesions, 33 participants (36.7%) had moderate lesions, and 13 participants (14.4%) had severe lesions.

**Table 1:** **General characteristics in the whole sample**.

	Whole sample (*N* = 90)
Age, M (SD)	80.0 (6.1)
Women% (*N*)	68.9 (62)
Educational level% (*N*)
Elementary school	11.0 (10)
Secondary school	26.8 (24)
High-school diploma and above	62.2 (56)
MMSE, M (SD)	27.0 (2.2)
Lacunes% (*N*)	16.7 (15)
MTL atrophy% (*N*)
None or light	21.1 (19)
Mild	32.2 (29)
Moderate	36.7 (33)
Severe	10.0 (9)
Subcortical WML% (*N*)
None	24.4 (22)
Light	47.8 (43)
Moderate or severe	27.8 (25)
Periventricular WML% (*N*)
None or light	48.9 (44)
Moderate	36.7 (33)
Severe	14.4 (13)

*% (*N*), percentage (number); M (SD), mean (standard deviation); MTL, medial temporal lobe; WML, white matter lesions*.

Considering vascular risk factors, 14 participants (15.5%) stated having experienced migraine, 31 were smokers (34.4%), 10 were above the at risk consummation of alcohol (11%), 44 had hypertension (48.9), 8 had diabetes (8.9%), 28 had high cholesterol levels (31.1%), 11 had coronary heart disease (12.2), 4 had chronic heart failure (4.4%), and 5 had atrial fibrillation (5.6%).

Correlations among personality traits are reported in Table 2. The mean value and standard deviation of each personality trait was 3.55 (0.71) for openness to experience, 3.55 (0.64) for conscientiousness, 3.13 (0.80) for extraversion, 4.14 (0.60) for agreeableness, and 2.83 (0.75) for neuroticism. Positive correlations were observed between openness to experience and conscientiousness (*p* < 0.05) and extraversion (*p* < 0.001). A positive correlation was also observed between conscientiousness and extraversion (*p* < 0.05). However, no correlation was observed between conscientiousness and neuroticism.

**Table 2:** **Means and correlation coefficients of personality traits**.

Personality traits	Whole sample	Kendall rank correlation coefficient				
	M (SD)	Openness	Conscientiousness	Extraversion	Agreeableness	Neuroticism
Openness	3.55 (0.71)	1.00				
Conscientiousness	3.55 (0.64)	0.16*	1.00			
Extraversion	3.13 (0.80)	**0.29^‡^**	0.18*	1.00		
Agreeableness	4.14 (0.60)	0.10	0.11	0.09	1.00	
Neuroticism	2.83 (0.75)	0.01	−0.10	−0.12	−0.06	1.00

M (SD), mean (standard deviation); *p < 0.05; ^‡^*p* < 0.001.

Table 3 reports correlations (Kendall) and comparisons (ANOVA) between the general characteristics according to personality traits. Age and MMSE score did not correlate with any of personality traits. Women had higher agreeableness scores than men (*F*_(1, 88)_ = 4.07; *p* < 0.05) and tended to have higher levels of neuroticism (*p* = 0.07). Openness to experience and agreeableness were associated with higher education level (*F*_(2, 87)_ = 4.36; *p* < 0.05; *F*_(2, 87)_ = 3.46; *p* < 0.05, respectively). Alcohol consumption was associated with lower extraversion (*F*_(1, 88)_ = 8.75; *p* < 0.01) and higher neuroticism (*F*_(1, 88)_ = 4.09; *p* < 0.05). Migraine episodes and smoking were associated with lower agreeableness (*F*_(1, 74)_ = 8.70; *p* < 0.01; *F*_(1, 88)_ = 4.06; *p* < 0.05, respectively). Hypertension was associated with lower openness to experience and extraversion (*F*_(1, 88)_ = 9.87; *p* < 0.01; *F*_(1, 88)_ = 10.63; *p* < 0.05, respectively). Finally, a trend between hypercholesterolemia and neuroticism was observed but it did not reach the significant level (*p* = 0.06).

**Table 3:** **General characteristics according to personality traits**.

Personality traits	Openness		Conscientiousness		Extraversion		Agreeableness		Neuroticism	
	M (SD) or τ	*p*	M (SD) or τ	*p*	M (SD) or τ	*p*	M (SD) or τ	*p*	M (SD) or τ	*p*
Age	−0.03	0.73	0.01	0.89	−0.05	0.52	0.13	0.09	−0.01	0.88
Sex
Men	3.66 (0.79)	0.30	3.66 (0.66)	0.28	3.05 (0.86)	0.53	**3.96 (0.53)**	<0.05*	2.61 (0.78)	0.07
Women	3.49 (0.67)		3.50 (0.64)		3.17 (0.77)		**4.23 (0.62)**		2.92 (0.73)	
Educational level
Primary	**2.94 (0.82)**	<0.05*	3.32 (0.92)	0.23	2.81 (0.91)	0.22	**3.76 (0.77)**	<0.05*	2.76 (0.84)	0.46
Secondary	**3.60 (0.64)**		3.44 (0.63)		3.01 (0.78)		**4.34 (0.45)**		2.99 (0.70)	
High-school diploma and above	**3.63 (0.68)**		3.64 (0.58)		3.24 (0.78)		**4.13 (0.61)**		2.77 (0.76)	
MMSE	0.10	0.22	0.11	0.15	0.10	0.18	−0.01	0.88	−0.06	0.41
Migraine	3.32 (0.98)	0.19	3.27 (0.73)	0.13	3.04 (0.71)	0.64	**3.70 (0.75)**	**<0.01^†^**	3.00 (0.90)	0.16
No migraine	3.60 (0.64)		3.57 (0.63)		3.16 (0.85)		**4.20 (0.53)**		2.68 (0.71)	
Ever smoker	3.64 (0.65)	0.39	3.44 (0.57)	0.22	3.06 (0.83)	0.57	**3.97 (0.56)**	<0.05*	2.91 (0.69)	0.45
Never smoker	3.50 (0.74)		3.61 (0.68)		3.16 (0.78)		**4.24 (0.61)**		2.78 (0.79)	
Alcohol consumption	3.41 (0.61)	0.51	3.27 (0.48)	0.15	**2.46 (0.72)**	**<0.01^†^**	4.18 (0.49)	0.86	**3.27 (0.75)**	<0.05*
No alcohol consumption	3.56 (0.72)		3.59 (0.66)		**3.21 (0.77)**		4.14 (0.62)		**2.77 (0.74)**	
Hypertension	**3.32 (0.80)**	**<0.01^†^**	3.46 (0.72)	0.19	**2.86 (0.82)**	**<0.01^†^**	4.20 (0.64)	0.42	2.96 (0.72)	0.11
No hypertension	**3.76 (0.53)**		3.64 (0.55		**3.38 (0.69)**		4.10 (0.57)		2.70 (0.77)	
Diabetes mellitus	3.23 (1.01)	0.19	3.78 (0.68)	0.30	2.64 (0.75)	0.07	4.08 (0.62)	0.75	3.00 (0.97)	0.49
No diabetes mellitus	3.58 (0.68)		3.53 (0.64)		3.18 (0.79)		4.15 (0.61)		2.81 (0.73)	
Hypercholesterol	3.44 (0.90)	0.34	3.53 (0.75)	0.86	3.10 (0.77)	0.80	4.08 (0.67)	0.47	3.04 (0.72)	0.06
No hypercholesterol	3.59 (0.61)		3.56 (0.60)		3.14 (0.81)		4.18 (0.57)		2.73 (0.75)	
Coronary heart disease	3.50 (0.81)	0.85	3.39 (0.63)	0.38	2.78 (0.90)	0.12	4.23 (0.55)	0.64	3.10 (0.55)	0.20
No coronary heart disease	3.55 (0.70)		3.57 (0.65)		3.18 (0.77)		4.14 (0.61)		2.79 (0.77)	
Chronic heart failure	3.46 (0.55)	0.82	3.38 (0.67	0.58	2.76 (1.22)	0.35	4.47 (0.48	0.27	2.99 (0.52)	0.66
No chronic heart failure	3.55 (0.72)		3.56 (0.65)		3.15 (0.78)		4.13 (0.61)		2.82 (0.76)	
Atrial fibrillation	3.24 (1.31)	0.33	3.45 (1.16)	0.71	2.73 (0.99)	0.25	4.30 (1.18)	0.56	2.75 (0.81)	0.82
No atrial fibrillation	3.56 (0.67)		3.56 (0.61)		3.15 (0.78)		4.14 (0.56)		2.83 (0.75)	

M(SD), Mean (Standard Deviation) comparison with ANOVA; τ, Kendall rank correlation coefficient; *p < 0.05; †p < 0.01.

Table 4 reports comparisons (ANOVA) between MRI abnormalities according to personality traits. The presence of lacunar stroke was not associated to any personality traits. Higher stages of MTL atrophy were associated with higher scores of openness to experience (*F*_(3, 86)_ = 3.24; *p* < 0.05). In contrast, higher stages of subcortical or periventricular WML were associated with higher scores in neuroticism (*F*_(2, 87)_ = 3.95; *p* < 0.05 and *F*_(2, 87)_ = 6.07; *p* < 0.01, respectively). Lower scores in conscientiousness were associated with subcortical (*F*_(2, 87)_ = 3.67; *p* < 0.05) but not with periventricular WML (*F*_(2, 87)_ = 2.66; *p* = 0.08). Finally, no other association was found between WML and the other personality traits (i.e., openness to experience, extraversion and agreeableness).

**Table 4:** **MRI abnormalities according to personality traits**.

Personality traits	Openness		Conscientiousness		Extraversion		Agreeableness		Neuroticism	
	M (SD)	*p*	M (SD)	*p*	M (SD)	*p*	M (SD)	*p*	M (SD)	*p*
Lacunes										
Yes	3.57 (0.81)	0.88	3.30 (0.82)	0.10	2.86 (0.90)	0.16	3.92 (0.65)	0.11	2.61 (0.93)	0.22
No	3.54 (0.78)		3.60 (0.63)		3.18 (0.84)		4.19 (0.59)		2.87 (0.70)	
Medial temporal atrophy										
None or light	**3.49 (0.54)**	<0.05*	3.32 (0.64)	0.22	2.89 (0.93)	0.21	4.18 (0.50)	0.79	2.65 (0.84)	0.39
Mild	**3.31 (0.76)**		3.52 (0.71)		3.08 (0.87)		4.05 (0.75)		3.02 (0.67)	
Moderate	**3.83 (0.67)**		3.70 (0.56)		3.35 (0.69)		4.19 (0.53)		2.77 (0.75)	
Severe	**3.36 (0.76)**		3.58 (0.67)		3.02 (0.46)		4.22 (0.61)		2.78 (0.83)	
Subcortical WML										
None	3.58 (0.57)	0.80	**3.82 (0.70)**	<0.05*	3.29 (0.69)	0.36	4.25 (0.56)	0.38	**2.46 (0.73)**	<0.05*
Light	3.49 (0.74)		**3.54 (0.55)**		3.14 (0.82)		4.17 (0.55)		**2.89 (0.69)**	
Moderate or severe	3.61 (0.80)		**3.33 (0.68)**		2.96 (0.84)		4.01 (0.72)		**3.04 (0.79)**	
Periventricular WML										
None or light	3.54 (0.76)	0.84	3.69 (0.63)	0.08	3.14 (0.76)	0.99	4.19 (0.60)	0.62	**2.59 (0.66)**	**<0.01**^†^
Moderate	3.51 (0.64)		3.36 (0.64)		3.12 (0.85)		4.15 (0.60)		**3.16 (0.70)**	
Severe	3.65 (0.75)		3.55 (0.61)		3.12 (0.84)		4.00 (0.67)		**2.78 (0.89)**	

MTL, medial temporal lobe; WML, white matter lesions; M (SD), mean (standard deviation) comparison with ANOVA; *p < 0.05; ^†^*p* < 0.01.

In the mixed model adjusted for age, sex, and education level (Table 5), lower scores of conscientiousness were associated with higher stages of subcortical (*p* < 0.01) but not with periventricular WML (*p* = 0.10) or MTL atrophy (*p* = 0.13). Similarly, levels of neuroticism were only associated with subcortical WML (*p* < 0.01).

**Table 5:** **Association of personality traits and lacunes, MTL atrophy, subcortical, and peripheral WML adjusted for age, sex, and education level**.

Personality traits	Openness			Conscientiousness			Extraversion			Agreeableness			Neuroticism		
	β (SE)	*p*	*p*_linear_	β (SE)	*p*	*p*_linear_	β (SE)	*p*	*p*_linear_	β (SE)	*p*	*p*_linear_	β (SE)	*p*	*p*_linear_
MTL atrophy															
None or light	Ref		0.36	Ref		0.13	Ref		0.24	Ref		0.85	Ref		0.91
Mild	−0.24 (0.29)	0.42		0.34 (0.29)	0.25		0.26 (0.29)	0.38		−0.19 (0.29)	0.51		0.50 (0.29)	0.09	
Moderate	0.45 (−0.29)	0.13		0.58 (0.29)	0.05		0.26 (0.29)	0.06		−0.01 (0.29)	0.98		0.13 (0.29)	0.64	
Severe	−0.23 (0.40)	0.56		0.36 (0.40)	0.38		0.11 (0.40)	0.78		0.02 (0.40)	0.97		0.11 (0.40)	0.78	
Subcortical WML															
None	Ref		0.99	**Ref**		**<0.01^†^**	Ref		0.11	Ref		0.12	**Ref**		**<0.01^†^**
Light	−0.15 (0.26)	0.57		−**0.46 (0.26)**	**0.07**		−0.21 (0.26)	0.41		−0.16 (0.26)	0.54		**0.54 (0.26)**	**0.04**	
Moderate or severe	−0.01 (0.29)	0.98		−**0.81 (0.29)**	**0.006**		−0.46 (0.29)	0.11		−0.44 (0.29)	0.13		**0.72 (0.29)**	**0.01**	
Periventricular WML															
None or light	Ref		0.90	Ref		0.10	Ref		0.72	Ref		0.26	Ref		0.09
Moderate	−0.06 (0.23)	0.78		−0.55 (0.23)	0.02		−0.06 (0.23)	0.79		−0.10 (0.23)	0.68		0.73 (0.23)	0.002	
Severe	0.09 (0.31)	0.78		−0.29 (0.31)	0.37		−0.10 (0.31)	0.75		−0.38 (0.31)	0.23		0.19 (0.31)	0.55	

*Mixed model for repeated measure and random intercept adjusted for age, sex, and education level. MTL, medial temporal lobe; WML, white matter lesions*.

*p < 0.05; ^†^*p* < 0.01.

In the mixed model adjusted for age, sex, and education level including MTL atrophy and subcortical and periventricular WML simultaneously (Table 6), lower scores of conscientiousness were associated with higher stages of subcortical WML (*p* < 0.01) but not with periventricular WML (*p* = 0.27) or MTL atrophy (*p* = 0.15). Higher scores of neuroticism were associated with higher stages of subcortical WML (*p* < 0.05) and periventricular WML (*p* < 0.05).

**Table 6:** **Association of personality traits and lacunes, MTL atrophy, subcortical, and peripheral WML, full adjusted model**.

Personality traits	Openness			Conscientiousness			Extraversion			Agreeableness			Neuroticism		
	β (SE)	*p*	*p*_linear_	β (SE)	*p*	*p*_linear_	β (SE)	*p*	*p*_linear_	β (SE)	*p*	*p*_linear_	β (SE)	*p*	*p*_linear_
MTL atrophy^a^															
None or light	Ref		0.40	Ref		0.15	Ref		0.27	Ref		0.89	Ref		0.87
Mild	−0.24 (0.29)	0.43		0.34 (0.29)	0.24		0.26 (0.29)	0.37		−0.19 (0.29)	0.52		0.51 (0.29)	0.09	
Moderate	0.43 (0.29)	0.14		0.56 (0.29)	0.06		0.55 (0.29)	0.06		−0.02 (0.29)	0.94		0.12 (0.29)	0.68	
Severe	−0.25 (0.40)	0.54		0.34 (0.40)	0.40		0.10 (0.40)	0.81		0.01 (0.40)	0.99		0.10 (0.40)	0.81	
subcortical WML^b^															
None	Ref		0.96	**Ref**		**<0.01**^†^	Ref		0.13	Ref		0.14	**Ref**		<0.05*
Light	−0.12 (0.26)	0.65		**−0.44 (0.26)**	**0.10**		−0.19 (0.26)	0.47		−0.13 (0.26)	0.61		**0.57 (0.26)**	0.03	
Moderate or severe	−0.03 (0.31)	0.93		**−0.83 (0.31)**	**0.009**		−0.48 (0.31)	0.12		−0.46 (0.31)	0.14		**0.70 (0.31)**	0.03	
Periventricular WML^c^															
None or light	Ref		0.59	Ref		0.27	Ref		0.93	Ref		0.24	**Ref**		<0.05*
Moderate	−0.003 (0.24)	0.99		−0.49 (0.23)	0.04		−0.001 (0.23)	0.99		−0.04 (0.24)	0.88		**0.79 (0.24)**	**0.001**	
Severe	0.22 (0.34)	0.51		−0.15 (0.34)	0.66		0.03 (0.34)	0.92		−0.25 (0.34)	0.47		**0.32 (0.34)**	**0.34**	

*^a^Mixed model for repeated measure and random intercept adjusted for age, sex, education level, and subcortical and peripheral WML*.

*^b^Mixed model for repeated measure and random intercept adjusted for age, sex, education level, MTL, atrophy, and peripheral WML*.

*^c^Mixed model for repeated measure and random intercept adjusted for age, sex, education level, MTL, atrophy, and subcortical WML*.

*MTL, medial temporal lobe; WML, white matter lesions*.

*p < 0.05; ^†^*p* < 0.01.

Sensitivity analyses were performed to take into account supplemental variables in addition to the variables already in the fully adjusted model. First, because alcohol consumption was associated with the score in neuroticism and a trend was observed between hypercholesterolemia and neuroticism, mixed models were adjusted for alcohol consumption and hypercholesterolemia. It did not modify the relationship between neuroticism and moderate and severe periventricular WML (*p* = 0.001 and *p* = 0.29, respectively) and between neuroticism and subcortical WML (*p* = 0.04 and *p* = 0.03, respectively). Second, because women tended to have higher scores in neuroticism, mixed models were run with BFI gender specific *z*-scores. It did not modify the relationship between neuroticism and moderate and severe periventricular WML (*p* = 0.001 and *p* = 0.26, respectively) and between neuroticism and light, moderate, or severe subcortical WML (*p* = 0.05 and *p* = 0.03, respectively). Finally, because migraine has been associated with scores in neuroticism, mixed models were adjusted for migraine. It did not modify the relationship between neuroticism and moderate and severe periventricular WML (*p* = 0.03), whereas it decreased the association between neuroticism and subcortical WML (*p* = 0.06).

## Discussion

In this study, involving an MCI population, lower levels of conscientiousness and higher levels of neuroticism were associated with the severity of WML. Meanwhile, presence of lacunar stroke and the severity of MTL atrophy were not associated with any personality traits.

Mild cognitive impairment patients are a population at high risk of AD (Gauthier et al., 2006), with a higher risk of conversion to AD for the amnestic MCI entity (Gauthier et al., 2006). A recent study evaluated personality traits in 63 MCI patients and 90 control subjects and shows, using caregiver ratings, that MCI subjects have higher scores of neuroticism and lower scores of other personality traits than controls (Donati et al., 2013). These changes of personality traits are similar to the ones described in early AD, which suggests that brain alterations responsible for this personality changes in MCI and AD patients share a common bases. However, the relationship between personality traits and structural brain changes has never been studied neither in MCI nor in AD patients. Indeed, no association between WML and personality traits has ever been reported so far in MCI subjects. Nevertheless, the association between personality traits and WML is in accordance with the literature in line with the cognitive consequences of these kind of cerebral lesions. First, WML are associated with depressive states in large transversal (Krishnan et al., 2006; Lee et al., 2012) and longitudinal studies (Godin et al., 2008; Firbank et al., 2012). Neuroticism is characterized by the tendency to experience distress and anxiety, along with difficulty in managing stress and controlling impulses (Costa and McCrae, 1987). Some of the characteristics defining neuroticism overlap with symptoms of depression and anxiety (Lahey, 2009). Consequently, several studies evaluated the relation between neuroticism and major depression (Kendler et al., 1993, 2006; Fanous et al., 2007) and strongly suggested that neuroticism has a predictive utility for depression. Second, WML are also associated with impairment in executive functions and lower processing speed (Bolandzadeh et al., 2012). Executive functions include working memory, selective attention, switching, and inhibition, and these cognitive processes underlie goal-directed and future-oriented behaviors (Alvarez and Emory, 2006). Deficits in these cognitive processes affect the ability to plan ahead and work steadfastly toward attaining goals, which are some features of the personality trait of conscientiousness. Indeed, conscientiousness is also called “will” (Digman and Inouye, 1986), “work” (Peabdy and Goldberg, 1990), or “dependability” (Fiske, 1949) and refers to the tendency to control impulses and be goal-directed (Wilson et al., 2007). Therefore, the present results highlight the association between WML and the dimension conscientiousness of personality. It is of note that periventricular WML were not associated with conscientiousness as much as subcortical WML. This may depend on the small sample size and therefore susceptible to a high inter-individual variability. It suggests that periventricular WML have less impact on the functioning of brain areas involved in conscientiousness than subcortical WML. Similarly, the association between periventricular WML and neuroticism were not as strong as with subcortical WML. Indeed, higher neuroticism was only associated with moderate periventricular WML, whereas it was associated with both moderate and severe subcortical WML. History of migraine episodes is associated with increased volume of white matter hyper-intensities (Hougaard et al., 2014) and neuroticism (Davis et al., 2013); an adjustment with this factor must decrease the association between neuroticism and WML. However, the fact that this association was still observed, clearly demonstrated that WML lesions are associated with neuroticism independently of migraine episodes. Nevertheless, personality traits associated with WML in this study are the same that the ones altered in patients suffering from multiple sclerosis, which induce white matter demyelination (Bruce and Lynch, 2011).

Altogether, these results provide new elements suggesting that WML alter the functioning of frontal areas. Neuroticism was recently associated with enhanced activity in the amygdala, anterior cingulate, medial prefrontal cortex, and hippocampus as well as with altered connectivity between these regions (Ormel et al., 2013). In addition, the personality trait conscientiousness seems associated with the activity of the prefrontal cortex but not with the activity of the hippocampus or the amygdala (DeYoung et al., 2010). Therefore, since WML are associated with both levels of neuroticism and conscientiousness, it is likely that such lesions alter the functioning of frontal area as also suggested by the impairment of executive functions (Alvarez and Emory, 2006).

The present study revealed that lacunar stroke was not associated with any of the personality dimensions. Although it is clearly established that cognitive impairment is common after lacunar strokes (for review, Makin et al., 2013), their associations with personality traits have not been previously studied.

The present study also revealed that MTL atrophy was not associated with scores of any of the personality traits. This was unexpected because an abundant body of literature clearly indicated that AD patients, who experience MTL atrophy, present decreased levels of conscientiousness and increased levels of neuroticism (for review, Robins Wahlin and Byrne, 2011). In addition, lower levels of conscientiousness are associated with early-stage of AD (77.5% of correct classification) (Duchek et al., 2007). In the present MCI population studied, only 10% of the subjects had a severe MTL atrophy, which could explain the lack of association with personality traits usually observed in AD patients displaying stronger MTL atrophy. It is also possible that the difference in the present results came from the use of self-reports. Duchek et al. (2007) reported that self-reports could differ from informant-reports on conscientiousness. Because of their perception of their memory impairment, patients could develop strategies to compensate deficits in everyday life, which can alter the score in conscientiousness. Indeed, Duchek et al. (2007) showed that AD patients consider themselves with similar levels of conscientiousness than healthy patients and not with lower levels of conscientiousness as informants do (Duchek et al., 2007). However, self-reports for neuroticism are reliable and give similar results as informant-reports (Duchek et al., 2007). In addition, higher neuroticism and lower conscientiousness were independently associated with the risk to develop AD using self-report questionnaire (Duberstein et al., 2011). Thus, the absence of association between conscientiousness, neuroticism, and MTL atrophy in the present study cannot result from self-reports utilization. It should be mentioned that the studies investigating personality changes in AD did not report on MRI abnormalities and thereby, do not provide direct comparisons with MTL atrophy and WML. Since in both studies (Duchek et al., 2007; Duberstein et al., 2011), the studied population was relatively old and similar to the one considered herein, it cannot be ruled out that changes in personality traits previously reported reflected WML effect rather than MTL atrophy alterations. Indeed, lower conscientiousness and higher neuroticism are associated with cerebrovascular disease, which is a risk factor for AD (Duron et al., 2012; Toledo et al., 2012). To support this idea, a large prospective study showed that history of depression increased the risk for AD but this risk is not mediated by smaller hippocampal or amygdalar volumes (Geerlings et al., 2008). Interestingly, the same authors showed more recently that depression is associated with widespread white matter atrophy (Geerlings et al., 2013). Altogether, it suggests that because of their association with cerebrovascular lesions, lower conscientiousness and higher neuroticism may be considered as risk factors for cognitive impairment, which can be predictive of AD (Gauthier et al., 2006). In addition, because WML seems to alter the functioning of frontal areas, associations between personality traits and executive functions should be studied in MCI subjects and AD patients.

The present study has some limitations. First, its cross-sectional design precludes causal inference. Second, the sample size was not large enough and could have weakened statistical differences, especially when correlations were studied with the fully adjusted model (i.e., between periventricular WML and conscientiousness). As mentioned in Section “Methods,” the different MCI subtypes [i.e., amnestic versus non-amnestic and single versus multiple domain (Petersen, 2004)] were not separately studied. Only a semi-quantitative evaluation of MTL atrophy was performed, and no volumetric assessment was provided. Finally, brain volume or cortical thickness was not evaluated.

The study has several strengths. It is the first study examining associations between personality traits and the different kind of MRI abnormalities in MCI subjects, revealing new information about the relation between these personality traits and the MRI abnormalities. Also, the multiple adjustments with several confounders limited the effect of unknown factors.

## Conclusion

In an MCI population, we reported associations between personality traits and WML but not with the presence of lacunar stroke and MTL atrophy. These results suggest that personality changes observed in a MCI population, at high risk of AD, are initially associated with WML, which can induce executive dysfunctions, rather than with MTL atrophy. This study emphasizes the need of a longitudinal study to determine if personality traits are associated with conversion to AD with regards to brain structural changes.

## Conflict of Interest Statement

The authors declare that the research was conducted in the absence of any commercial or financial relationships that could be construed as a potential conflict of interest.
